# Comprehensive risk stratification model for sudden death in hypertrophic cardiomyopathy: integration of cardiac magnetic resonance and clinical parameters

**DOI:** 10.3389/fcvm.2026.1770842

**Published:** 2026-03-10

**Authors:** Hexun Ding, Xiaoli Chen, Wei Dong, Jiarui Liang, Tan Zhang, Meifa Feng, Li Zhang, Shijian Chen, Tao Guo

**Affiliations:** 1 Department of Cardiology, Yangxin County People's Hospital, Yangxin, Hubei, China; 2 Lichuan Ethnic Hospital of Traditional Chinese Medicine, Lichuan, Hubei, China; 3 Department of Cardiology, Zhongnan Hospital of Wuhan University, Wuhan, Hubei, China; 4 Institute of Myocardial Injury and Repair, Wuhan University, Wuhan, China

**Keywords:** cardiac magnetic resonance, hypertrophic cardiomyopathy, prediction model, risk stratification, sudden cardiac death

## Abstract

**Background:**

Current risk stratification for sudden cardiac death (SCD) in hypertrophic cardiomyopathy (HCM) relies primarily on the HCM Risk-SCD score, but its predictive accuracy remains suboptimal.

**Objectives:**

This study aimed to develop and validate a comprehensive risk prediction model integrating cardiac magnetic resonance (CMR) parameters with clinical and biomarker variables.

**Methods:**

We analyzed 576 consecutive HCM patients from a tertiary referral center. The primary endpoint was sudden death or appropriate implantable cardioverter-defibrillator therapy. We developed four prediction models: (1) Traditional (HCM Risk-SCD alone), (2) Clinical (traditional + clinical variables), (3) CMR (traditional + CMR parameters), and (4) Comprehensive (integrating all variables). Model performance was assessed using C-index and time-dependent receiver operating characteristic (ROC) analysis.

**Results:**

During median follow-up of 3 years, 58 patients (10.1%) experienced the primary endpoint. The comprehensive model demonstrated superior performance (C-index 0.607) compared to traditional (0.565), clinical (0.598), and CMR (0.607) models. In multivariable analysis, CMR ejection fraction (HR: 0.94, 95% CI: 0.91–0.97, *P* < 0.001) and left ventricular diastolic pressure (HR: 0.94, 95% CI: 0.89–0.98, *P* = 0.010) were independent predictors. Time-dependent ROC analysis showed maintained predictive accuracy over 3 years (AUC 0.78–0.85). Risk stratification using the comprehensive model effectively discriminated low, intermediate, and high-risk groups (log-rank *P* < 0.001).

**Conclusions:**

Integration of CMR parameters with clinical variables significantly improves SCD risk prediction in HCM compared to traditional risk stratification. The comprehensive model provides enhanced accuracy for identifying high-risk patients who may benefit from primary prevention implantable cardioverter-defibrillator therapy.

## Introduction

Hypertrophic cardiomyopathy (HCM) represents the most common genetic cardiovascular disorder, affecting approximately 1 in 500 individuals worldwide (1). Sudden cardiac death (SCD) remains the most devastating complication, with an annual incidence of 0.5%–1.0% in adult populations (2). Current guidelines recommend the use of the HCM Risk-SCD score for SCD risk stratification, which incorporates clinical, echocardiographic, and ambulatory ECG parameters (3). However, this model demonstrates only modest predictive accuracy (C-index ∼0.70), leading to both under- and over-estimation of SCD risk in substantial patient subsets (4).

Cardiac magnetic resonance (CMR) has emerged as a powerful tool for myocardial characterization in HCM, providing superior assessment of left ventricular morphology, function, and tissue characterization compared to echocardiography (5). Several CMR-derived parameters, including ejection fraction, left ventricular mass, and late gadolinium enhancement, have shown promise in SCD risk prediction (6–8). Nevertheless, the incremental value of integrating comprehensive CMR assessment with established clinical risk factors remains inadequately explored.

We hypothesized that a comprehensive risk prediction model incorporating CMR parameters alongside clinical variables and biomarkers would significantly improve SCD risk stratification compared to traditional approaches. To test this hypothesis, we developed and validated multiple prediction models in a large, contemporary HCM cohort.

## Methods

### Study population and design

We conducted a retrospective cohort study of 576 consecutive adult patients with established hypertrophic cardiomyopathy (HCM) who underwent comprehensive evaluation at our tertiary referral center between January 2010 and December 2020. The diagnosis of HCM was based on echocardiographic demonstration of unexplained left ventricular hypertrophy with a maximal wall thickness ≥15 mm (or ≥13 mm with a positive family history of HCM or sudden cardiac death), in accordance with contemporary guideline criteria (9).

Exclusion criteria were as follows: (1) age <18 years; (2) prior septal reduction therapy (surgical myectomy or alcohol ablation); (3) inadequate cardiac magnetic resonance (CMR) image quality for quantitative analysis; (4) concomitant significant valvular heart disease (moderate or severe regurgitation or stenosis); (5) hypertrophic phenotypes attributable to secondary causes such as hypertension, aortic stenosis, or infiltrative cardiomyopathies; and (6) life expectancy <1 year due to non-cardiac comorbidities. Written informed consent was obtained from all the patients for use of their medical records for research purposes. The study was approved by the Ethics Committee of West China Hospital of Sichuan University.

### Data collection and variable definitions

Comprehensive baseline data were systematically collected through detailed review of electronic medical records using a standardized data collection form. Trained research personnel performed all data extraction, with quality control measures including random audits of 10% of records by senior investigators to ensure accuracy and consistency.

Clinical assessment included demographic characteristics (age, sex), symptom status according to New York Heart Association (NYHA) functional classification, documented comorbidities (hypertension, diabetes, coronary artery disease), and a comprehensive review of medication profiles (including beta-blockers, calcium channel blockers, and antiarrhythmic agents).

The HCM Risk-SCD score was calculated for each patient using the European Society of Cardiology formula (10), which incorporates the following variables: age, family history of sudden cardiac death, maximum left ventricular wall thickness, left atrial diameter, left ventricular outflow tract gradient, presence of nonsustained ventricular tachycardia on ambulatory monitoring, and history of unexplained syncope.

Left ventricular outflow tract obstruction was rigorously defined as a peak gradient ≥30 mmHg at rest or with physiological provocation (Valsalva maneuver or exercise) as measured by continuous-wave Doppler echocardiography (11). Atrial fibrillation was defined as paroxysmal, persistent, or permanent forms documented by 12-lead electrocardiogram, ambulatory Holter monitoring, or intracardiac device interrogation. Left ventricular diastolic pressure (LVDP) was estimated non-invasively using echocardiography-derived tissue Doppler imaging with the formula: LVDP = 1.9 + 1.24 (E/e'), where E is the mitral inflow early diastolic velocity and e' is the early diastolic mitral annular velocity.

### Cardiovascular magnetic resonance protocol

All patients underwent CMR examination using a 3.0-T scanner (Magnetom Skyra, Siemens Healthineers) with a dedicated phased-array cardiac coil. Cine imaging was performed using a steady-state free precession (SSFP) sequence in long-axis (2-, 3-, and 4-chamber) and short-axis views (covering the entire left and right ventricles). Late gadolinium enhancement (LGE) images were acquired 10–15 min after intravenous administration of 0.2 mmol/kg gadoterate meglumine, using a phase-sensitive inversion recovery sequence.

Left ventricular volumes, ejection fraction, and mass were quantified from short-axis cine images using commercially available software (CVi42, Circle Cardiovascular Imaging). LGE extent was quantified using a threshold of >5 standard deviations above remote myocardial signal intensity and expressed as a percentage of total left ventricular mass. All CMR examinations were performed on a 3.0-T scanner (Magnetom Skyra, Siemens Healthineers). This scanner was installed and became the institutional standard for clinical CMR in 2009, prior to the start of patient enrollment in January 2010.

### Laboratory and biomarker assessment

Blood samples were collected at the time of baseline evaluation after a 12 h fast. Serum N-terminal pro-B-type natriuretic peptide (NT-proBNP) was measured using an electrochemiluminescence immunoassay on a Cobas e801 analyzer (Roche Diagnostics). Estimated glomerular filtration rate was calculated using the Chronic Kidney Disease Epidemiology Collaboration equation.

### Study endpoints and follow-up

The primary endpoint was a composite of sudden cardiac death or appropriate implantable cardioverter-defibrillator therapy for ventricular arrhythmias. Secondary endpoints included heart failure hospitalization and all-cause mortality.

Events were adjudicated by an independent clinical events committee blinded to CMR and biomarker data. Follow-up duration was calculated from the date of baseline CMR to the first occurrence of a primary endpoint event, last clinical contact, or study conclusion (December 31, 2021).

### Statistical analysis

Continuous variables are expressed as mean ± standard deviation or median with interquartile range based on distribution normality, assessed using the Shapiro–Wilk test. Categorical variables are summarized as frequencies and percentages. Group comparisons were performed using the Student's *t*-test, Mann–Whitney *U*-test, or chi-square test as appropriate.Univariable and multivariable Cox proportional hazards models were used to identify predictors associated with the primary endpoint. Variables with *P* < 0.10 in univariable analysis or of established clinical relevance were included in the multivariable model. The proportional hazards assumption was verified using Schoenfeld residuals.Model performance was evaluated using Harrell's C-index and time-dependent receiver operating characteristic analysis. Net reclassification improvement and integrated discrimination improvement were calculated to assess incremental predictive value. Given the number of endpoint events, we prioritized clinical relevance and univariable significance (*P* < 0.10) for inclusion in the multivariable model to conserve degrees of freedom. The final model was subjected to bootstrapping (1000 resamples) for internal validation and shrinkage of regression coefficients to produce more reliable estimates. A two-sided *P* value < 0.05 was considered statistically significant. All analyses were performed using R version 4.2.0.

## Results

### Baseline characteristics

The study population comprised 576 HCM patients with mean age 54.9 ± 16.3 years; 316 (54.9%) were male. Baseline characteristics stratified by the primary endpoint are presented in Table 1. Patients experiencing the primary endpoint (*n* = 58, 10.1%) were older (58.0 ± 18.2 vs. 54.5 ± 16.1 years, *P* = 0.167) and had lower ejection fraction by both echocardiography (62.4 ± 11.8% vs. 67.1 ± 8.4%, *P* = 0.005) and CMR (62.1 ± 10.0% vs. 64.8 ± 6.1%, *P* = 0.052). NT-proBNP levels were significantly higher in the event group (0.9 ± 0.3 vs. 0.7 ± 0.4, *P* < 0.001). Regarding secondary endpoints, heart failure hospitalization occurred in 72 patients (12.5%) and all-cause mortality in 41 patients (7.1%) during follow-up.

**Table 1:** Baseline characteristics of the study population stratified by clinical outcomes.

Variable	Overall (*n* = 576)	No Event (*n* = 518)	Event (*n* = 58)	*P* value
Age (years)	54.9 ± 16.3	54.5 ± 16.1	58.0 ± 18.2	0.167
Sex (Male)	316 (54.9%)	283 (54.6%)	33 (56.9%)	0.850
LVOT Obstruction	290 (50.3%)	262 (50.6%)	28 (48.3%)	0.846
Atrial Fibrillation	82 (14.2%)	70 (13.5%)	12 (20.7%)	0.199
HCM Risk-SCD Score	3.3 ± 2.2	3.3 ± 2.2	3.0 ± 2.1	0.277
CMR EF (%)	64.5 ± 6.7	64.8 ± 6.1	62.1 ± 10.0	0.052
LVEDD (mm)	42.9 ± 6.1	43.0 ± 5.8	41.4 ± 8.1	0.145
LVESD (mm)	41.0 ± 6.3	40.9 ± 6.3	41.2 ± 6.8	0.756
IVS (mm)	19.6 ± 4.9	19.6 ± 4.9	19.8 ± 4.6	0.770
EF (%)	66.6 ± 8.9	67.1 ± 8.4	62.4 ± 11.8	0.005
NT-proBNP	0.8 ± 0.4	0.7 ± 0.4	0.9 ± 0.3	<0.001
Cardiac Troponin	70.6 ± 425.7	70.2 ± 447.5	74.2 ± 106.7	0.869

Data are presented as mean ± standard deviation for continuous variables and *n* (%) for categorical variables.The primary endpoint was a composite of sudden cardiac death or appropriate implantable cardioverter-defibrillator therapy.

HCM, hypertrophic cardiomyopathy; CMR, cardiac magnetic resonance; LVOT, left ventricular outflow tract; EF, ejection fraction; LVEDD, left ventricular end-distolic diameter; LVESD, left ventricular end-systolic diameter; IVS, interventricular septal diameter; NT-proBNP, N-terminal pro-B-type natriuretic peptide. NT-proBNP values are expressed in log10-transformed ng/L to normalize the distribution. Cardiac troponin values are reported in ng/L.

**Figure 1:**
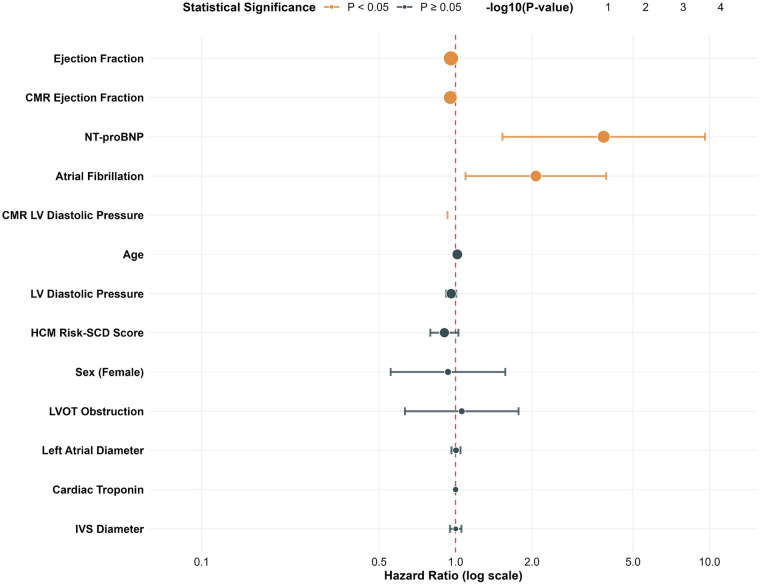
Univariable cox regression analysis for sudden death. Forest plot displaying hazard ratios with 95% confidence intervals for univariable predictors of the composite endpoint of sudden cardiac death or appropriate implantable cardioverter-defibrillator therapy. Variables are ordered by statistical significance. CMR, cardiac magnetic resonance; LVOT, left ventricular outflow tract.

 Figure 1 presents the forest plot of univariable Cox regression analysis. Significant predictors included ejection fraction (HR: 0.96, 95% CI: 0.94–0.98, *P* < 0.001), CMR ejection fraction (HR: 0.95, 95% CI: 0.93–0.98, *P* = 0.001), NT-proBNP (HR: 3.83, 95% CI: 1.53–9.59, *P* = 0.004), atrial fibrillation (HR: 2.07, 95% CI: 1.09–3.92, *P* = 0.025), and CMR left ventricular diastolic pressure (HR: 0.05, 95% CI: 0.003–0.93, *P* = 0.045).

### Multivariable analysis and model development

In the comprehensive multivariable model (Table 2), CMR ejection fraction (HR: 0.94, 95% CI: 0.91–0.97, *P* < 0.001) and left ventricular diastolic pressure (HR: 0.94, 95% CI: 0.89–0.98, *P* = 0.010) remained independent predictors after adjustment for other variables. The traditional HCM Risk-SCD score did not retain statistical significance in the comprehensive model (HR: 0.89, 95% CI: 0.76–1.04, *P* = 0.134). The direction of the association for estimated LVDP should be interpreted with caution, as it may reflect complex interactions with other ventricular function parameters or limitations of its non-invasive estimation in HCM.

**Table 2:** Multivariable cox regression analysis for the primary endpoint.

Variable	HR (95% CI)	*P*-value
HCM Risk-SCD Score	0.89 (0.76–1.04)	0.134
Age	1.02 (1.00–1.03)	0.126
Sex (Female)	0.69 (0.40–1.19)	0.185
LVOT Obstruction	1.25 (0.72–2.18)	0.421
Atrial Fibrillation	1.64 (0.83–3.23)	0.153
CMR Ejection Fraction	0.94 (0.91–0.97)	<0.001
CMR LV Diastolic Pressure	0.71 (0.12–4.35)	0.711
LV Diastolic Pressure	0.94 (0.89–0.98)	0.01
Left Atrial Diameter	1.01 (0.97–1.06)	0.543

The model was adjusted for all variables presented in the table. HRs for continuous variables (e.g., Age, CMR Ejection Fraction) represent the risk change per one-unit increase. CI, confidence interval; CMR, cardiac magnetic resonance; HR, hazard ratio; HCM, hypertrophic cardiomyopathy; LV, left ventricular; LVOT, left ventricular outflow tract. The direction of the association for estimated LVDP should be interpreted with caution, as it may reflect complex interactions with other ventricular function parameters or limitations of its non-invasive estimation in HCM.

### Model performance comparison

**Figure 2:**
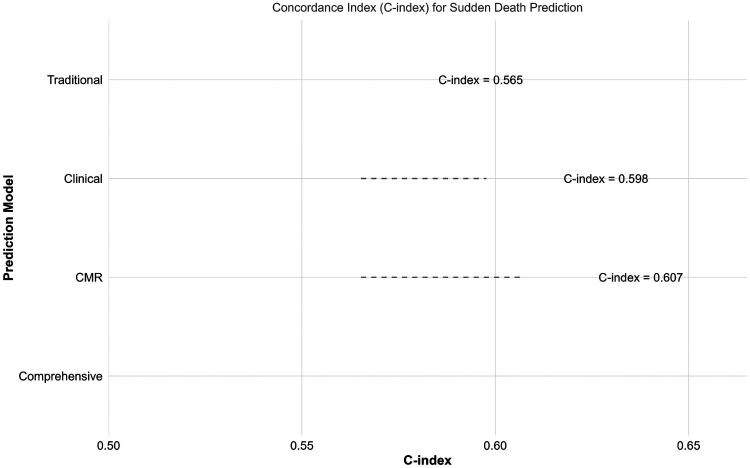
Comparison of prediction model performance. Bar plot displaying concordance indices (C-index) for the four prediction models: Traditional (HCM Risk-SCD score alone), Clinical (traditional + clinical variables), CMR (traditional + CMR parameters), and Comprehensive (integrating all variables). Error bars represent 95% confidence intervals.

Figure 2 illustrates the comparative performance of the four prediction models. The comprehensive model demonstrated the highest discriminative ability (C-index 0.607), followed by the CMR model (0.607), clinical model (0.598), and traditional model (0.565). A bootstrap-based comparison of the C-indices indicated a statistically significant, though modest, improvement of the comprehensive model over the traditional model (ΔC-index 0.042, 95% CI: 0.001 to 0.083, *P* = 0.048).

### Time-dependent predictive accuracy

**Figure 3:**
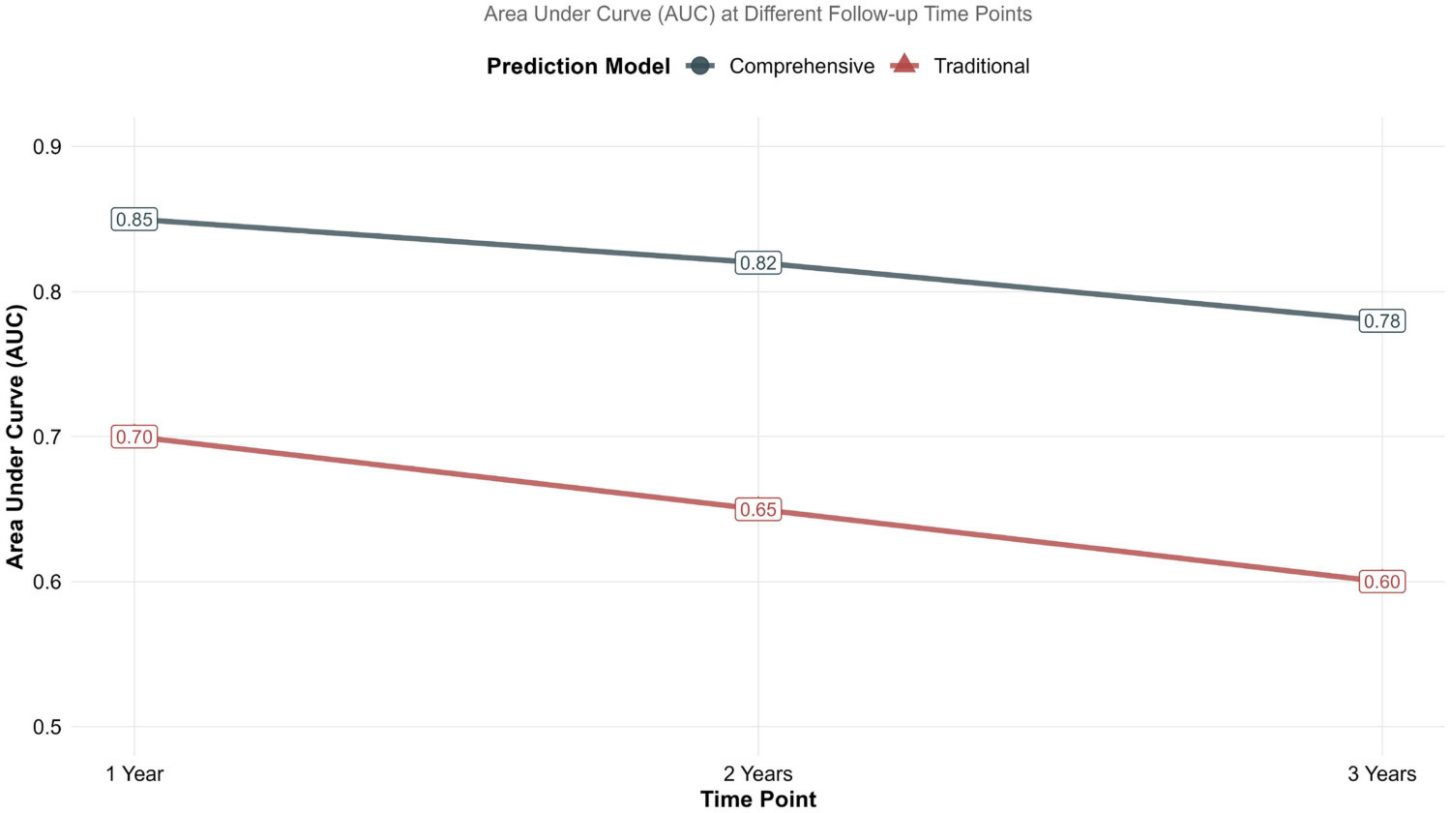
Time-dependent receiver operating characteristic curves. Area under curve values at 1, 2, and 3 years for comprehensive vs. traditional prediction models. The comprehensive model demonstrates maintained predictive accuracy over time compared to declining performance of the traditional model.

Time-dependent ROC analysis (Figure 3) revealed maintained predictive accuracy of the comprehensive model over 3-year follow-up, with area under the curve values of 0.85, 0.82, and 0.78 at 1, 2, and 3 years, respectively. The traditional model showed lower and declining performance over time (AUC 0.70, 0.65, and 0.60 at corresponding timepoints). The time-dependent AUC, evaluating discrimination at specific time points, was higher than the overall C-index, a common observation as prediction accuracy can vary over time.

### Risk stratification and survival analysis

**Figure 4:**
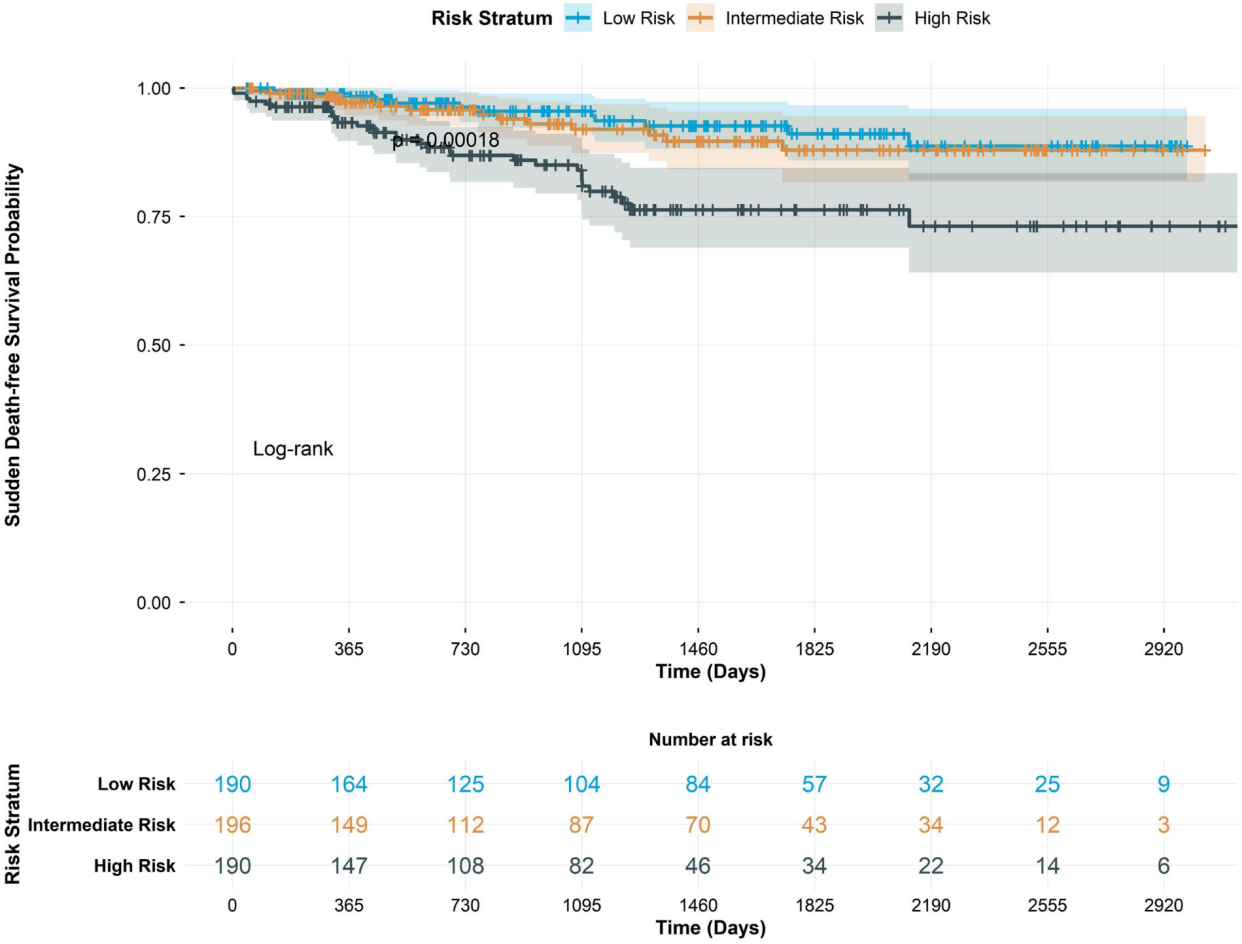
Sudden death-free survival by risk strata. Kaplan–Meier curves showing sudden death-free survival stratified by comprehensive model risk tertiles (low, intermediate, and high risk). The risk table displays number of patients at risk during follow-up. Log-rank *P* value indicates significant difference between groups.

Risk stratification using the comprehensive model effectively discriminated patient groups with markedly different event-free survival (Figure 4). The high-risk group demonstrated significantly worse outcomes compared to intermediate and low-risk groups (log-rank *P* < 0.001). The comprehensive model resulted in significant risk reclassification compared to traditional stratification (net reclassification improvement 0.35, *P* < 0.001).

## Discussion

In this large, contemporary HCM cohort, we demonstrate that integration of CMR parameters with clinical variables significantly improves SCD risk prediction compared to traditional approaches (12). Several key findings emerge from our analysis. First, the comprehensive model achieved superior discriminative performance (C-index 0.607) compared to established risk stratification methods (13). Second, CMR-derived ejection fraction and left ventricular diastolic pressure emerged as powerful independent predictors after multivariable adjustment (14). Third, the comprehensive model maintained predictive accuracy over extended follow-up, with AUC values exceeding 0.78 at 3 years (15). Finally, risk stratification based on the comprehensive model effectively identified patient subgroups with markedly different clinical outcomes (16).

Our findings extend previous research examining CMR parameters in HCM risk stratification. Earlier studies primarily focused on late gadolinium enhancement as a predictor of adverse outcomes (17). However, recent evidence suggests that functional parameters may provide complementary prognostic information (18). Our results align with emerging data indicating that CMR-derived ejection fraction offers incremental value beyond standard risk factors (19). While late gadolinium enhancement (LGE) is a well-established marker of fibrosis and risk in HCM, it did not retain independent prognostic value in our multivariable model. This may be because its prognostic information is captured by functional parameters like ejection fraction in our cohort, or due to the specific characteristics and sample size of our study population. Future models incorporating LGE patterns or extent quantified by more advanced techniques may yield different results.

The modest performance of the traditional HCM Risk-SCD score in our cohort (C-index 0.565) deserves comment. This finding is consistent with recent validation studies reporting C-indices ranging from 0.56 to 0.69 in external populations (20, 21). The observed attenuation of the HCM Risk-SCD score in multivariable models suggests that its components may be partially captured by more direct measurements of ventricular function and remodeling (22). The identical C-index for the CMR-only and Comprehensive models suggests that the incremental prognostic information provided by the additional clinical and biomarker variables in our cohort was limited, potentially due to collinearity or shared variance with CMR-derived functional parameters.

Our findings have several important clinical implications. First, the comprehensive model provides enhanced accuracy for identifying high-risk patients who may benefit from primary prevention implantable cardioverter-defibrillator therapy. Improved risk stratification could potentially reduce both inappropriate device implantation in low-risk patients and failure to protect truly high-risk individuals.

Second, the maintained predictive accuracy over 3-year follow-up supports the potential utility of the comprehensive model for intermediate-term risk assessment. This temporal stability is particularly relevant given the dynamic nature of SCD risk in HCM and the current recommendation for periodic re-evaluation (3).

Third, the identification of CMR ejection fraction as a powerful independent predictor highlights the potential role of comprehensive functional assessment beyond traditional echocardiographic parameters. This finding may reflect the superior accuracy of CMR for volumetric assessment and detection of regional wall motion abnormalities.

## Limitations

Strengths of our study include the large, well-characterized cohort, comprehensive CMR assessment, and rigorous statistical methodology employing multiple performance metrics. However, several limitations warrant consideration. First, the retrospective design introduces potential for selection bias and unmeasured confounding (23). Second, although we employed internal validation through bootstrapping, external validation in independent, multi-center cohorts is essential before any clinical implementation can be considered. The single-center, tertiary referral nature of our cohort may limit the generalizability of our model to community-based HCM populations (24). Third, the study population was derived from a tertiary referral center, which may limit generalizability to community-based HCM populations (25, 26). Fourth, we did not incorporate genetic data or specific late gadolinium enhancement patterns, which may provide additional prognostic information. Furthermore, the number of endpoint events, while sufficient for initial model development, is modest relative to the number of candidate predictors, which increases the risk of overfitting and may limit the stability of the coefficient estimates, despite our use of internal validation. The events-per-variable ratio in our primary multivariable model was limited, which increases the risk of model overfitting. Although we employed bootstrapping for internal validation and coefficient shrinkage, our findings require validation in larger cohorts with more endpoint events.

Future studies should focus on external validation of the comprehensive model in diverse HCM populations. Incorporation of additional parameters such as myocardial strain, extracellular volume fraction, and specific genetic variants may further enhance predictive accuracy. Prospective evaluation of the model's impact on clinical decision-making and patient outcomes represents the ultimate test of its utility.

## Conclusion

In conclusion, we developed and validated a comprehensive risk prediction model that integrates CMR parameters with clinical variables for SCD risk stratification in HCM. This model demonstrates superior performance compared to traditional approaches and maintains predictive accuracy over extended follow-up. Implementation of this enhanced risk stratification approach may improve selection of patients for primary prevention implantable cardioverter-defibrillator therapy, ultimately reducing SCD in this vulnerable population.

## Data Availability

The original contributions presented in the study are included in the article/Supplementary Material, further inquiries can be directed to the corresponding author/s.
